# Potential of Natural Fiber Reinforced Polymer Composites in Sandwich Structures: A Review on Its Mechanical Properties

**DOI:** 10.3390/polym13030423

**Published:** 2021-01-28

**Authors:** S. Alsubari, M. Y. M. Zuhri, S. M. Sapuan, M. R. Ishak, R. A. Ilyas, M. R. M. Asyraf

**Affiliations:** 1 Advanced Engineering Materials and Composites Research Centre (AEMC), Department of Mechanical and Manufacturing Engineering, University Putra Malaysia, Serdang 43400, Malaysia; snsubari@gmail.com (S.A.); sapuan@upm.edu.my (S.M.S.); 2 Laboratory of Biocomposite Technology, Institute of Tropical Forestry and Forest Product (INTROP), University Putra Malaysia, Serdang 43400, Malaysia; mohdridzwan@upm.edu.my; 3 Department of Aerospace Engineering, University Putra Malaysia, Serdang 43400, Malaysia; asyrafriz96@gmail.com; 4 Aerospace Malaysia Research Centre (AMRC), University Putra Malaysia, Serdang 43400, Malaysia; 5 Sustainable Waste Management Research Group (SWAM), School of Chemical and Energy Engineering, Faculty of Engineering, University Teknology Malaysia, Johor Bahru 81310, Malaysia; ahmadilyas@utm.my; 6 Centre for Advanced Composite Materials (CACM), University Teknologi Malaysia, Johor Bahru 81310, Malaysia

**Keywords:** core structure, honeycomb, mechanical properties, natural fiber, polymer composites, sandwich structure

## Abstract

The interest in using natural fiber reinforced composites is now at its highest. Numerous studies have been conducted due to their positive benefits related to environmental issues. Even though they have limitations for some load requirements, this drawback has been countered through fiber treatment and hybridization. Sandwich structure, on the other hand, is a combination of two or more individual components with different properties, which when joined together can result in better performance. Sandwich structures have been used in a wide range of industrial material applications. They are known to be lightweight and good at absorbing energy, providing superior strength and stiffness-to-weight ratios, and offering opportunities, through design integration, to remove some components from the core element. Today, many industries use composite sandwich structures in a range of components. Through good design of the core structure, one can maximize the strength properties, with a low density. However, the application of natural fiber composites in sandwich structures is still minimal. Therefore, this paper reviewed the possibility of using a natural fiber composite in sandwich structure applications. It addressed the mechanical properties and energy-absorbing characteristics of natural fiber-based sandwich structures tested under various compression loads. The results and potential areas of improvement to fit into a wide range of engineering applications were discussed.

## 1. Introduction

Sandwich structure is known as a core attached to two identifiable stiff and strong skins; the core distributes the load from one skin to another. While the skins contribute to the bending and turgidity, the adhesive that conveys the shear and axial loads to the core material is used as a bonding agent [1,2]. The skin (or sometimes called a face sheet), and which in rare cases weighs only a few millimeters, is composed of light alloys. These light alloys range from aluminum and single metallic layer to laminated or fiber-reinforced composites. In sandwich structures, core designs such as honeycomb, lattice, truss, web-reinforced, and cellular are commonly used. Opting for particular sandwich materials is largely dependent on a number of factors, which include the structure’s use [3], lifetime loading [4], and the value and price of the materials [5,6]. For aerospace applications, carbon epoxy and graphite epoxy with multilayered facings are used. However, for the facings of civil, marine, and household products, glass epoxy or glass vinyl ester is utilized [7,8].

According to Gay, Hoa, and Tsai [9], aerospace structures use a core made of aluminum or Nomex honeycomb. Birman and Kardomateas [10] suggested that civil engineering requires the use of a closed-cell or open-cell foam, while ship sandwich structures require the use of balsa. Therefore, these combinations of material have resulted in a competitive structure in various application areas, such as aerospace, transportation, marine, and civil structures, where high strength, low weight materials, and fuel economy are essential factors; as well as impact mechanics and high-energy absorbing materials. A comprehensive study of energy absorption and crashworthiness was published by Carruthers et al. [11]. This review demonstrated that fiber reinforced plastics can be designed to exhibit higher normalized energy absorption capabilities than the metals that have been traditionally used for vehicle construction. Alghamdi [12] reviewed research on the common shapes of collapsible energy absorbers, such as circular and square tubes, frusta, struts, honeycombs, and sandwich plates. A study on impact mechanics and high energy absorbing structures and materials, as well as new concepts for design structures, such as the high energy absorbing properties of lattices structure, was published by Qiao et al. [13]. Chai and Zhu [14] reviewed the low-velocity impact of sandwich structures. The mechanical response of composite sandwich structures with tubular inserts to quasi-static compression was studied by Tarlochan et al. [15]. As noted by Zuhri et al. [16], sandwich structures can be seen in natural fiber structures such as bamboo and grass.

Recently, natural fiber reinforced composites have been used in various applications due to their low cost, light weight, high strength-to-weight ratio, renewability, low density, and energy requirements for processing, etc. However, notwithstanding, with certain environments and load requirements, their limitations constrain their application. To address the issue of natural fibers’ inferior load requirement and mechanical properties, fiber treatment and hybridization have received significant attention by researchers. Via these methods, researchers are able to design a sandwich structure of minimal water absorption and optimum mechanical performance. Here, the potential use of natural fiber materials in a sandwich structure application is comprehensively studied. This review will focus on the current studies of sandwich structure materials, and the potential for using natural fiber composites in sandwich structure applications. Furthermore, the mechanical performance of cellular foams, and corrugated and honeycomb cores under quasi-static and dynamic compression loadings will be discussed. In addition, this review looks inward to how a sandwich structure made of natural fiber composite yielded improved performance, e.g., energy-absorbing characteristics. The article will feature different types of hybrid sandwich structure, with their performances, limitations, and possible areas of improvement for use in a wide range of applications.

## 2. Natural Fiber Composites: Background, Properties, and Applications

The utilization of natural fibers as alternative materials in many industrial sectors has been of major global interest, for moving towards a greener environment and sustainability. These natural fibers, e.g., kenaf, sugar palm, flax, jute, hemp, etc. have been incorporated with polymeric resins to form new materials, called natural fiber composites (NFCs) [17,18,19,20,21]. These natural fibers can be mixed with bioplastics such as PLA, phenolics, and starch to produce green composites [22,23,24,25,26,27]. In general, these NFCs are constituted from thermoset or thermoplastic resins to offer specific qualities and properties for various applications [28,29,30,31]. From this point of view, it can be seen that the application of NFCs has grasped the attention of material researchers and engineers due to the advantages they offer (Table 1) [32]. On top of this, the current environmental issues have expedited the growing interest among industries for innovating with NFCs, for various products.

From the aforementioned statements, NFCs have been suggested to substitute for the previous traditional composites, including glass, basalt, carbon, and aramid fiber composites [32]. This could be happening due to the NFCs themselves, which have low manufacturing costs, with higher productivity, as well as good mechanical strength and stiffness. Generally, the performance of NFCs depends on the fiber type. For instance, bast fibers tend to show superior flexural strength, while leaf fibers tend to offer excellent impact properties [33]. Thus, the understanding of natural fiber properties and forms is crucial for achieving successful improvement outcomes [34,35,36,37]. Chandrasekar et al. [38] found that flax fiber reinforced epoxy composites exhibited higher bending and impact strengths. Of equal importance, an accurate aspect ratio should be considered, because natural fibers can exist in different forms, such as entangled yarn, bundles, and elementary fiber [39]. There are various factors that influence the mechanical performances of a natural fiber, e.g., fiber content, temperature, humidity, fiber treatment, and fiber type.

**Figure 1:**
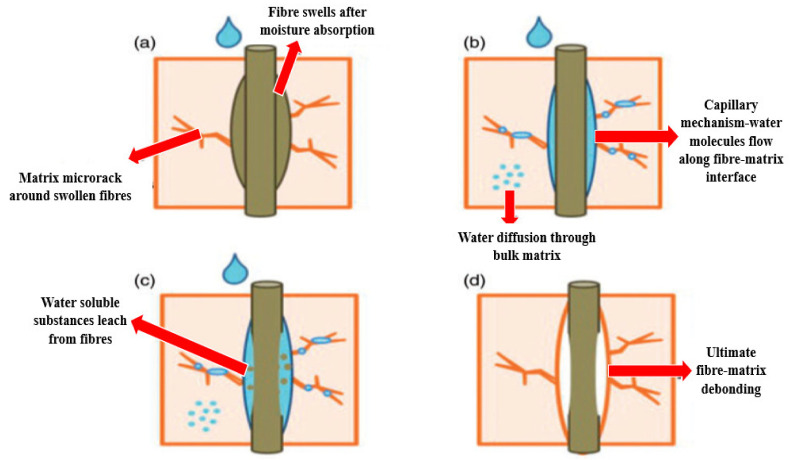
Effect of water absorption of natural fiber on mechanical properties of composite laminate:(**a**) fibre swells after moisture absorption,(**b**) capillary mechanism water molecules flow along fibre-matrix interface, (**c**) water soluble substance leach from fibres, and (**d**) ultimate fibre-debonding [43].

Typically, natural fibers such as flax and hemp have a high potential for substituting the traditional synthetic fibers. On the other hand, natural fibers are considered challenging materials, since their mechanical properties vary widely, depending on their source, storage method, and nature [40,41]. The most prominent issues during the fabrication of NFCs include poor water resistance, low durability, and poor fiber–matrix adhesion. The poor interfacial adhesion between natural fibers and their polymer matrix is because of natural fibers’ composition; hemicellulose, pectin, and lignin [32,42]. These components exhibit hydrophilicity, whereby a matrix is usually hydrophobic, causing the natural fiber filler to be easily removed, cracked, fractured, and cratered. Moreover, this phenomenon happens due to fibers and matrices that are not strongly bonded with each other. Figure 1 briefly explains how the high moisture absorption of natural fibers diminishes the overall mechanical performance of NFCs. The lack of interfacial bonding, and the absorption of moisture, cause NFCs to be less efficient for stress transfer, and to have low durability.

NFCs also have thermal degradation problems, as the composite laminate is exposed to overheating. To solve the issue, fiber treatment is one of the methods that can be considered to improve the surface topography of fibers for industrial uses. These treatments provide a better interlocking system between fiber and matrix due to good surface roughness and fiber–matrix compatibility [31]. Improvements in interfacial bonding automatically improve the overall performance of natural fiber composites. Subsequently, the NFCs can fulfill a wider range of applications. There are three main fiber treatments for improving the quality of NFCs: alkali, silane, and acetyl treatments [44]. Alkali treatment, or mercerization, aids the removal of lignin and hemicellulose [42], whereas silane treatment provides a coating layer on the surface of the natural fibers [45]. For acetylation, the mercerization process is carried out to remove the hemicellulose, natural fats, lignin, and waxes from the acetylating cellulose [46]. In this case, the process can yield a higher potential of hydroxyl groups and other reactive functional groups on the fibers’ surface. Subsequently, the NFCs are comparable with glass fiber composites, especially in impact strength properties.

**Figure 2:**
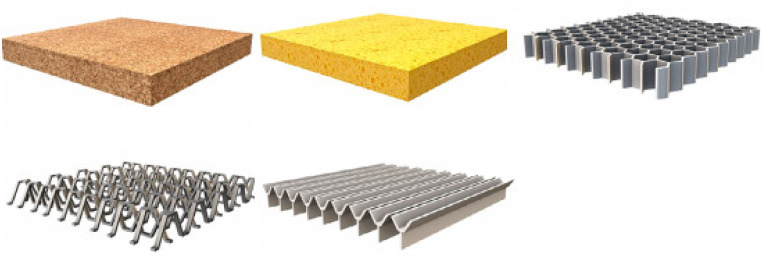
Examples of core shapes for sandwich structures [51].

Still, the progress of NFCs has continued to snowball since efforts for promoting the recyclability of crop wastes into goods to replace synthetic fibers were initiated. Natural fibers are highly promising for application as sandwich structures, either as the face sheet sandwich, or the inside core structure. They can replace the aluminum honeycomb, NOMEX, glass fibers, and other traditional materials in sandwich structures, which are highly useful in aerospace, automobile, civil, and marine applications [7]. These NFCs are produced by hand lay-up, compression molding, and vacuum bagging techniques to attain good, finished sandwich products. The application of NFCs in sandwich structures could provide better mechanical properties, and move resistance performance towards more extended durability [47,48,49,50]. Figure 2 displays examples of core shapes for sandwich structures.

## 3. Sandwich Core Structure

There are several types of sandwich cores that have been studied in recent years. All studies were targeting the development of a lightweight structure with good strength and stiffness. Today, researchers are seeking an archetypal lightweight cellular core that can be used in sandwich structures, e.g., stochastic and periodic cells. Gibson and Ashby [52] suggested that foam (open or closed cells), classified as a random microstructure, is one of the most-used stochastic materials. On the other hand, Wadley [53] asserted that the periodic structures produced from a chain of unit cells in an array could be grouped into three categories; honeycomb structures, prismatic topologies, and lattice truss structures. Table 2 shows the classification of cellular materials as cores for sandwich structures.

### 3.1. Cellular Foams

A number of techniques can be used in foaming various forms of solids. According to Liu and Chen [55], the phases of foaming polymer foams include bubble nucleation, expansion, and solidification. Ashby et al. [56] stated that metal foams that have solid metals in them are usually produced from aluminum with gas pores. Cellular foams with pores can be open- or closed-cells. Gong and Kyriakides [57] explained that cellular materials are beneficial because of their ease of manufacturing, and for alternating the densities by a small percent (~2–10%) of the base material’s density. Detailed studies on the manufacturing process of cellular foams have been extensively performed by many researchers [55,58,59,60]. Though metal foams have higher density than polymer foams, they are similar in the sense that they can sustain large compressive strains, and can be used in energy-absorbing applications.

Extensive studies have been performed on the energy-absorbing capability of cellular foam-based core structures. An investigation of the compressive response of three polymeric foam materials, i.e., expanded polystyrene (EPS), high-density polyethylene (HDPE), and polyurethane (PU), under quasi-static testing (medium and high strain rate conditions), was made by Ashraf et al. [1]. They discovered that EPS and HDPE materials showed an increasing value of crush stress plateaus and a decreasing strain value when the strain rate was increased. They also exhibited an increasing value of material density. The PU material, on the other hand, displayed large-scale fractures and emission at intermediate rates. This caused a depletion in the strength of the crush plateau in comparison with low rate tests.

Subhash et al. [61] examined the function of polymeric structural foams with varying porosity levels, and low density (<1 g/cm^3^) and high density (>1 g/cm^3^), under low and high strain rate loadings. It was found that an increase in foam density also led to an increase in Young’s modulus, yield strength, maximal stress, and strain to failure, while the high-density foams showed an increasing consistency in deformity, and resulted in a ductile-like fracture form, the low-density foams failed as a result of the prior disintegration of large porous cells. In addition, Saha et al. [62] carried out a test on two polymeric foams; cross-linked poly-vinyl chloride (PVC) and polyurethane (PUR). This was done under compression loading at varying strain rates. The analysis showed that peak stress and energy absorption were largely dependent on the microstructure, density, foam materials, and strain rate.

**Figure 3:**
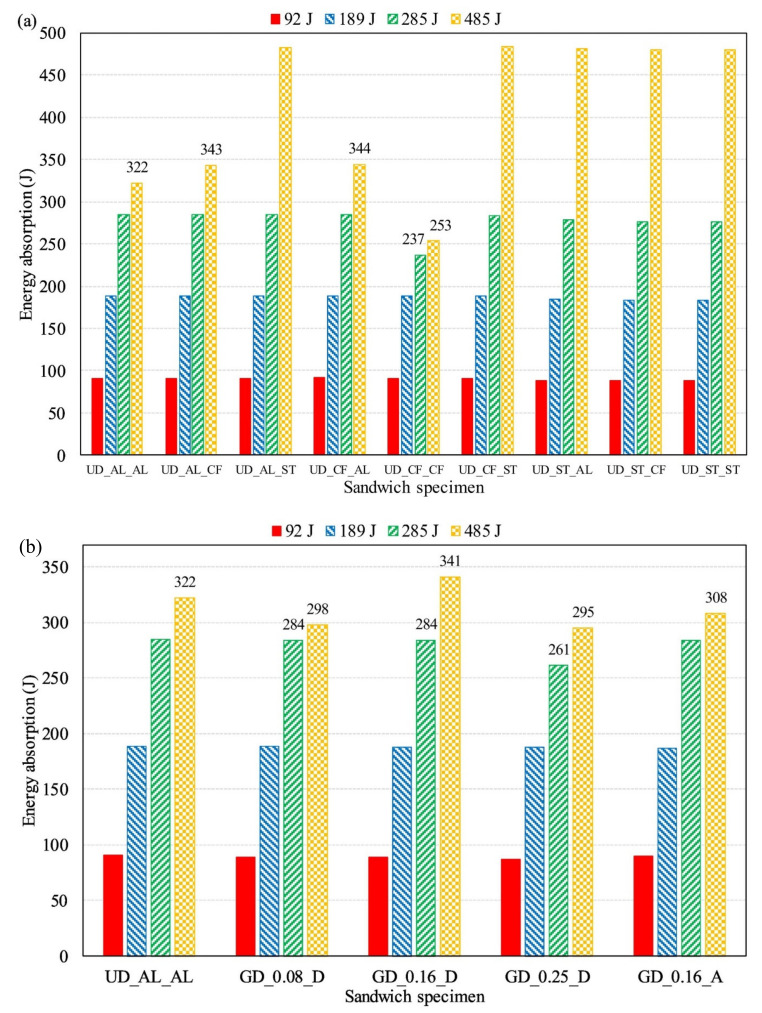
Energy absorption of sandwich panels under different impact energies: (**a**) sandwich panels with different face sheets, (**b**) sandwich panels with homogeneous and various graded foam cores [64].

Chen et al. [63] investigated several advanced foam-filled multi-cell composite panels (FMCPs) made from glass fiber-reinforced polymer (GFRP) skins, GFRP lattice webs, and polyurethane (PU), under quasi-static compression. This study investigated four parameters: the effects of the skin and lattice-web thickness, the foam-cell width and height, and the foam density. The analysis showed great reductions in the peak crushing force and bearing loads for the FMCP with trapezoidal cells. Importantly, the FMCP with double-layer dislocation cells exhibited a specific energy absorption capacity and the highest mean crushing load. A study on the response of sandwich panels made of uniform (UD) and several graded density (GD) foam cores and skins produced from aluminum (AL), stainless steel (ST), and carbon fiber (CF) was conducted by Sun et al. [64]. The UD and CF were noted as having the highest specific energy absorption in comparison with the other panels, while specimens produced with GD_0.16_D were recorded as having the highest energy absorption when compared with the other panels, with less than 485 J impact, as shown in Figure 3.

### 3.2. Corrugated Core

Recent studies have shown that tremendous attention is being given to metallic sandwich structures, which are now regarded as protective structures in fields such as aerospace, marine, and transportation [65,66]. This is because the plastic deformation absorbs the impact energy in a situation where the structure suffers from an unexpected crash. Several sandwich structures with various cores, such as foams [67], honeycomb [54], and truss [68], were recommended by scholars for making the structures more crashworthy. Dayyani et al. [69] proposed that corrugated structures would have an impact in engineering applications. This is because they have exceptional structural characteristics, including extreme anisotropic behavior, and a high stiffness to weight ratio, resulting from their geometric properties.

Several experimental and numerical studies on the quasi-static or dynamic behavior of sandwich structures have been carried out. A trial of an aluminum honeycomb-corrugation hybrid core sandwich construction was performed by Han et al. [70]. This led to the combination of aluminum honeycomb with corrugated aluminum that resulted in appreciably enhanced compressive strength and absorption of energy, mainly in the low-density regime. Zhang et al. [71] explored the effect of varying thicknesses on the dynamic behaviors of sandwich steel plates and graded corrugated cores. They considered three different core arrangements of sandwich plates with identical core density: BBBB, AACC, and ABBC, where A referred to 0.762 mm, B referred to 0.508 mm, and C referred to 0.254 mm. The results and elucidation revealed that the ABBC absorbed the smallest plastic energy in the back substrate, and the most in the core. In addition, Hou et al. [72] carried out a number of experimental studies and numerical simulations on multilayered corrugated sandwich panels under quasi-static crushing loading conditions. The study concluded that there was an extension of the plateau stage, as well as that the addition of more layers led to an increasing value of energy absorption before densification.

A comparison of the specific compressive response of composite corrugation cores, conventional honeycomb, and foam materials was investigated by Rejab and Cantwell [73]. They reported that the compressive components of thick corrugated cores were like those of aluminum honeycomb. Furthermore, Yan et al. [74] noted increases in the compressive strength and energy absorption capacity of a hybrid sandwich by 211% and 300%, respectively. Meanwhile, an increase in value for specific energy absorption by 157% was caused by a foam filling into the empty corrugated sandwich core. Similarly, Damghani and Gonabadi [75] assessed the role of foam-core relative density on the impact properties of sandwich panels. They discovered that an increase in the foam-core would invariably lead to a rise in the impact resistance and energy absorption. In addition, Yang [76] reported that specific energy absorption was partly dependent on the thickness of the corrugated core’s layer. Moreover, a study on scaling effects in the compression response of sandwich structures was carried out by Zhou et al. [77]. In the study, the authors used both carbon and glass fibers, as well as varying the corrugation thickness and the number of unit cells. The results showed that the tested cores had no notable scaling effects, and as the corrugation got thicker, the compression strength was increased.

### 3.3. Honeycomb

Honeycomb refers to a lightweight structure that requires a number of cells to be fused together to form a structure. Aluminum, Nomex, or thermoplastics, such as propylene, are examples of materials used for honeycomb structures [78]. As previously reported, it was shown that hexagonal honeycomb structures offered an outstanding mechanical performance, allowing them to be used as sandwich core structures [79,80]. Furthermore, as noted by Côté et al. [81], the presence of bonded skins affected the compressive response of the core with a relative density of 0.20, more than the cores with 0.03 and 0.10 relative densities. They also made a comparison between steel square honeycomb and aluminum hexagonal honeycomb. The hexagonal honeycomb showed lower peak stress and increased rapid softening, and exceeded the peak load when compared with the square honeycomb.

**Figure 4:**
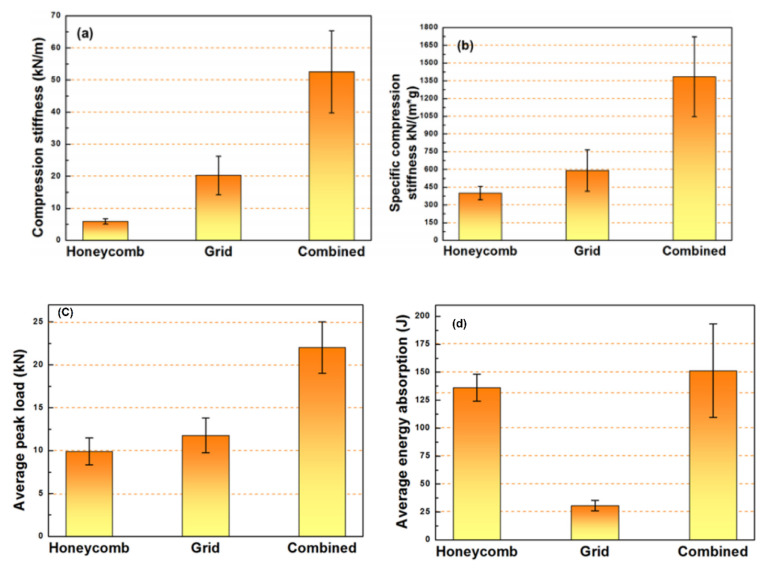
Average properties of sandwich specimens: (**a**) compression stiffness, (**b**) specific compression stiffness, (**c**) peak load, and (**d**) energy absorption [82].

In addition, the compressive properties of composite sandwich structures with a grid reinforced honeycomb core were studied by Sun et al. [82]. Here, they used a honeycomb core, a grid core, and a combination of the two cores. The report showed that the combination of the cores exhibited an increased stiffness, as well as better energy absorption and critical load. This can be observed when comparing it with the honeycomb core and the grid core individually (see Figure 4).

**Figure 5:**
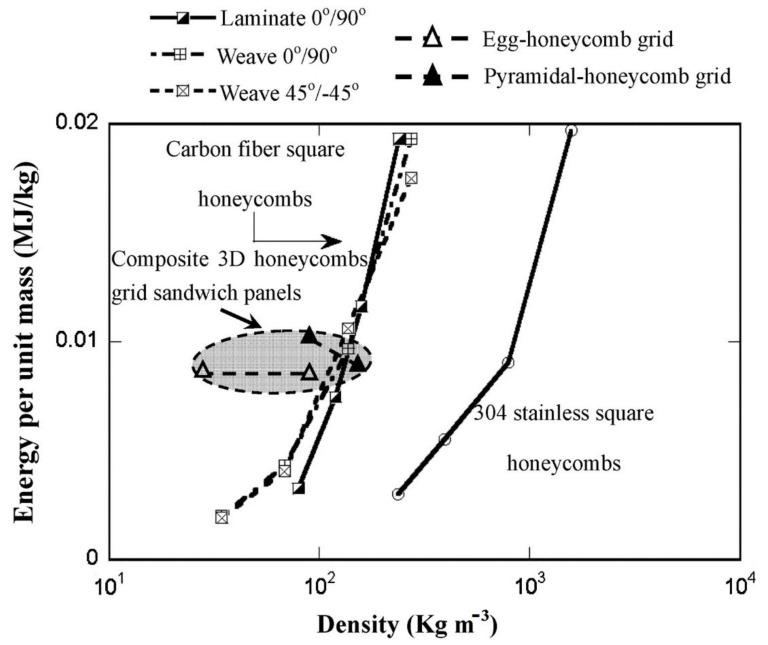
A comparison between the energy absorption capacities per unit mass of competing metallic and composite grids sandwich panels, as reported in [83].

Furthermore, Xiong et al. [83] also examined an egg honeycomb grid core and a pyramidal honeycomb grid core regarding their compression and energy absorption. An assessment of these carbon fiber reinforced honeycomb cores showed that the advanced honeycomb core exhibited higher energy absorption than the lightweight square honeycombs of equal density, as shown in Figure 5.

## 4. Fabrication Process of Core Structure

Table 3 presents the details of fabrication processes for composite cores. It can be seen that a sandwich core structure can be fabricated from NFCs to produce good quality and more robust products. Even so, the fabrication of a NFC core is quite challenging. To ensure the NFC cores are well fabricated, parameter settings must be optimized to eliminate any possible defects. In general, injection molding and compression molding are among the suitable manufacturing techniques to fabricate the core of the sandwich structure. An NFC core structure is not suitable for being fabricated using injection molding due to the low melt flow index of the fabrication process. Recently, Azaman et al. [84] developed a thin-walled structure using NFCs via injection molding, which has potential to be used to manufacture the core for a sandwich structure. Some studies have also reported that a NFC core could have various issues, such as porosity, and uneven wall thickness.

Even though NFC core technology is still in an infant stage, regarding identifying its capabilities, many studies have been conducted on manufacturing NFCs cores using the compression molding technique to solve the porosity issues. The main disadvantages of fabricating via compression molding are de-bonding of the cell wall interface, buckling of the cell wall, and fracture. Stocchi et al. [92] conducted research on the comparison of NFC honeycomb core properties from two different manufacturing processes. Jute fiber-reinforced vinyl ester composites were used to manufacture honeycomb cores from molds with fixed inserts and lateral compression. The honeycomb core fabricated from lateral compression had higher performance, due to a technique that removed excess resin and air. Conversely, the lateral compression technique entailed additional time to produce the hexagonal cores. Rao et al. [94] found that sinusoidal cores and hexagonal cores from sisal fiber-reinforced polypropylene composites were higher than commercial cores using compression molding and roll forming methods, in terms of specific modulus and strength. Table 4 depicts the summary of the specific mechanical properties of the sisal–polypropylene core.

In addition, the fabrication of flax fiber-reinforced linear low-density polyethylene (LLDPE) composites was conducted via compression molding to form a corrugated shape [89]. The process was initiated by a layering process of dry flax fiber mat and LLDPE film in molding slots, and ended with a compression action at 170 °C. However, Rao et al. [94] implemented an extrusion process to produce a better corrugated sheet than the previous compression molding technique. This was due to the increase in edge constraints. Apart from those mentioned techniques, a flax fiber-reinforced polylactic acid (PLA) composite honeycomb core was also manufactured using the slotting method. In the first place, the composite was prepared by the lamination process to form a sheet. Later, the square and triangular honeycombs were produced by the slotting technique. The research was supported by FEA results showing that progressive failure can happen within the honeycomb design slots. Thus, the fabrication technique of NFC cores plays a significant role in producing a higher quality of sandwich structure.

## 5. Mechanical Properties of NFCs Core Sandwich Structures

The study of natural fiber composites has reached the point where natural fiber can be a viable option to replace glass fiber, with high specific strength and modulus [95]. Natural fiber composites in engineering applications have increased in recent years due to their advantages over conventional mineral fiber composites. The benefits include low density, high specific strength, increased toughness compared to glass fiber, increased sound absorption, improved biodegradability, and being environmentally cleaner and rapidly renewable [96]. Several natural fiber reinforced polymer composites have been studied recently for use in sandwich structure applications. Natural fiber composite is used as both a skin and core material for sandwich panel constructions. For example, Du et al. [97] investigated sandwich panels containing a paper reinforced polymer (PRP) composite as skin material, and natural fiber incorporated into the structure as a core. The authors claimed that the cell size and height of the honeycomb core greatly affected the mechanical properties of the sandwich structure. The findings indicated that the sandwich structure studied here could replace the existing commercial products. In a recent work, Du et al. [98] produced bio-based sandwich-structured composites, with both skin and core materials made from a biofiber and polylactic acid (PLA) matrix. Subsequently, an assessment of the flexural properties and failure modes of the structures was carried out. The study’s results showed that 50% of the fiber had the highest compressive properties, and thus, the flexural properties and failure mode of the composites met the automotive load floor requirements. Stocchi et al. [92] fabricated a novel honeycomb core made of jute-reinforced vinylester composite, manufactured by lateral compression molding. In contrast to the commercially available cores, the developed cores possessed high compression strength. Furthermore, Petrone et al. [89] developed an eco-friendly honeycomb core for sandwich panels by combining flax fiber with a polyethylene matrix; the analyses involved both reinforced and un-reinforced cores. The authors reported a significant improvement for reinforced cores (continuous-unidirectional and short-random) compared to un-reinforced ones in terms of the mechanical properties. In addition, an improvement in damping value was also achieved by filling the core with wool fiber, resulting in minimal weight increase. More works and investigations on the mechanical behavior of natural fiber were reported in [99].

**Figure 6:**
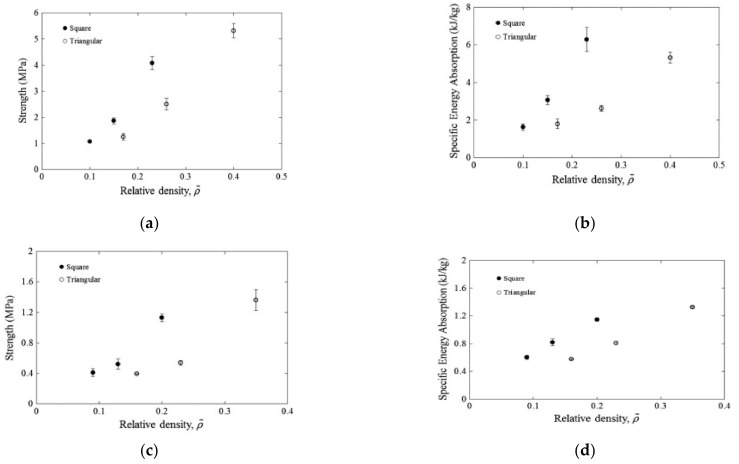
The compression strength and energy absorption of (**a**,**b**) flax/PP and (**c**,**d**) flax/PLA interlocking structures [88].

Manipulating the core material and its design can change the behavior of the sandwich structure, both in terms of compression and impact properties. For example, Jusoh et al. [100] stated that more experimental works should be conducted on corrugated core sandwich structures made of natural fibers. It is evident that, based on the information in the literature, the type of core material can significantly alter the overall properties of the composite. This can be seen in recent work done by Zuhri et al. [88], where two different types of flax-based honeycomb core were compared. The core materials were flax/polypropylene (flax/PP) and flax/polylactide (flax/PLA); the results showed that a sandwich structure with a flax/PP core offered higher strength and excellent energy-absorbing characteristics over those offered by the flax/PLA core, (see Figure 6).

Furthermore, a comparison of the capacity of bamboo and foam as core materials was investigated by Zuhri et al. [101]. The results revealed that the energy absorbing capacity of the bamboo structure increased as the diameter-to-thickness ratio was decreased. Moreover, the combination of a bamboo–foam composite structure offered higher energy absorption capacity than the sum of the individual foam and bamboo, which was due to the constraint of the foam. In a similar study, eco-friendly corrugated cores made of flax fiber combined with a polyethylene matrix were studied subject to a low-velocity impact [89]. The authors reported that continuous fiber reinforcement polymers gave better results than short fiber composites in response to impact loading. The presence of a skin increased the energy absorption because of energy dissipation. Petrone et al. [89] studied polymeric honeycombs reinforced with short and continuous flax fiber. They concluded that the energy absorption was increased with the core thickness, and the effect of the presence of skin on the energy absorption was minimal. In addition, sisal fiber reinforced with a polymer matrix, in the form of core structures, was investigated by Rao et al. [94]. The design of the core significantly affected the properties of the sandwich structure. Similar results were found in a study by Roslan et al. [102], who carried out a compression test on square and triangular honeycomb core structures based on a bamboo/epoxy composite. The different designs of the cores had a varying effect of the specific energy absorption. Therefore, it was evident, based on the data, that the core made of natural fiber-reinforced composites could offer an excellent result. On the other hand, Alsubari et al. [103] used flax fiber to prepare a square interlocking core with a double cell wall to investigate their compression properties under quasi-static loading. The results of the study showed significant enhancements in strength and energy absorption capacity, by approximately 80 and 86%, respectively, compared to single-cell wall cores reported in the literature. Table 5 summarizes selected papers on the development of cores from natural fiber.

Natural fibers have various origins, and as such, their characteristics differ, and can disallow their use in recent structures. According to Nishino et al. [105], factors that determine their properties, such as the shape, strength, and size of the natural fibers, include their place of origin, maturity, retting process, and the cultivation environment. These variations will also cause complexity in the properties of the natural fiber composites. The variation in the characteristics of NFCs means that each of them have their own strengths and weaknesses. Hence, a proper design that involves the hybridization of two different cellulosic fibers portends excellent enhancement of the properties of the hybrid composite. Due to their cellulosic nature, this type of hybrid fiber composites provide more flexibility and serve as a more economical choice [106]. Despite their advantages, some factors might hinder natural fibers’ performance during the hybridization process. These include fiber loading, fiber selection, fiber form, fiber orientation, fiber arrangement, matrix selections, porosity, and interfacial strength [107,108,109,110,111,112].

The hybridization of NFCs to improve their mechanical properties has become an area of interest to researchers in recent times. For instance, Alavudeen et al. [113] presented a study on the mechanical properties of kenaf/banana hybrid composites. It was shown that the kenaf/banana composites offered better mechanical properties compared to the individual fiber-based composites. In a similar study by Venkatesh et al. [114], the addition of bamboo fiber to sisal-unsaturated polyester composites enhanced their mechanical properties compared to the sisal-unsaturated polyester composites on their own. Moreover, Wu et al. [115] hybridized silk fiber with flax fiber, and studied their mechanical properties. The study showed that the hybrid exhibited improved flexural strength and impact strength over other NFCs. In addition, an examination of the mechanical properties of sisal and banana fiber-reinforced polylactide acid (PLA) composite was carried out by Gupta et al. [116]. According to their study, the treated fibers gave better mechanical properties than pure PLA and untreated fiber bio-composites.

In recent times, a few numbers of studies have been carried out on the hybridization of natural fibers in composite manufacturing, with respect to their energy absorption characteristics. For instance, an examination of an external basalt layers’ influence on the mechanical degradation of flax composites, when situated in critical environments, was performed [117]. The authors reported that the external basalt laminate, when added to a flax composite, ensured improved mechanical stability in the static and dynamic loads. For flax-based composites, they were able to increase their energy absorption capability with increased aging time, meanwhile the hybrid of basalt and flax was unable to vary its impact strength significantly. Similarly, Živković et al. [118] analyzed the effect of moisture absorption on the impact properties of basalt, flax, and hybrid of flax/basalt fiber composites under both dry and conditioned states. The authors observed significant energy-absorption improvements for the hybrid composites compared to their single composite counterparts, especially in conditioned samples. In addition, Senthil Kumar et al. [119] examined the hybridization of banana (B) and coconut (C), and the effect of their layering pattern. The individual composites and their hybrids were compared, and the results showed that having the coconut fiber as the outer layer exhibited the best damping behavior and therefore, a better energy absorption capability.

In addition, Wu et al. [115] investigated a hybrid of flax and silk fiber-reinforced composites. It was reported that when compared with the individual composites, this hybridization increased the energy absorption. In another study, it was found that hybridization of ramie/jute fiber-reinforced composites with a volume fraction of ramie fiber, about 55%, improved the energy absorption and impact resistance compared to individual fibers [120].

## 6. Conclusions

Natural fiber reinforced polymer composite materials are replacing synthetic materials to a great extent due to their eco-friendly, non-toxic, and biodegradable nature. However, there are certain issues associated with these fibers that need to be tackled to enhance their properties and workability. This review paper explored the potential use of natural fiber-based composites in sandwich structure applications. A brief overview of the classification of cellular materials as cores for sandwich structures, e.g., cellular foams, corrugated, and honeycomb cores, was given. An assessment of the individual fibers, as well as the combination of two fibers, was made. Moreover, the mechanical behavior of the fibers under static and dynamic loads was also discussed. As noted above, a number of studies have focused on enhancing the performance of both synthetic fibers, and hybrid synthetic with natural fibers, under dynamic and static loads. However, these researchers called for more studies on sandwich structures made from natural fibers, and a mix of natural with synthetic fibers, regarding their energy absorption capability. This is because they possess outstanding structural properties. The hybridization of natural fibers exhibits exceptional properties, and helps to preserve environmental resources. Researchers in recent times have focused on examining the hybridization of natural and synthetic composites since they could minimize cost and weight, as well as offering outstanding structural properties. In summary, it is recommended that more research should be done to decipher the behavior of natural fiber sandwich composites in engineering applications.

## Figures and Tables

**Table 1:** Benefits and drawbacks of natural fiber composites (NFCs).

Benefits	Drawback
Very good sound, acoustic, and electrical insulating properties	Moisture absorption, causing fibers to swell
Reactivity-materials provide sites for water absorption, and are also available for chemical modification	Restricted maximum processing temperature
Biodegradability: as a result of their tendency to absorb water, natural fibers will biodegrade under certain circumstances through the actions of fungi and\or bacteria	Lower durability, fiber treatments can improve this drawback
Combustibility: products can be disposed of through burning at the end of their useful service life, and energy can simultaneously be generated	Dimensional stability as a consequence of the hygroscopicity of fibers, products, and materials
Very good mechanical properties, especially tensile strength. In relation to their weight, the best fibers attain strength similar to Kevlar.	Variability in quality, dependent on unpredictable variables such as weather
The abrasive nature of natural fibers is much lower compared to glass fibers, which leads to advantages in regards to the technical aspects, material recycling, or processing of composites materials	Less fire retardance
Plant fibers are renewable raw materials and their availability is unlimited	Lower strength properties, particularly impact strength

**Table 2:** Classification of cellular materials as sandwich structures cores [54].

Cellular Materials					
Periods				Stochastic	
Three Dimensions (Lattice)		Two Dimensions			
Truss	Textile	Honeycombs	Prismatic	Open-cell	Closed-cell
Pyramidal ** 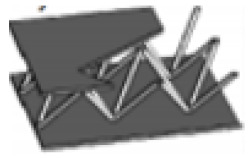 **	Diamond Collinear 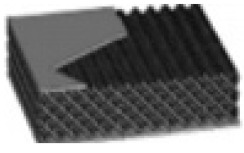	Square 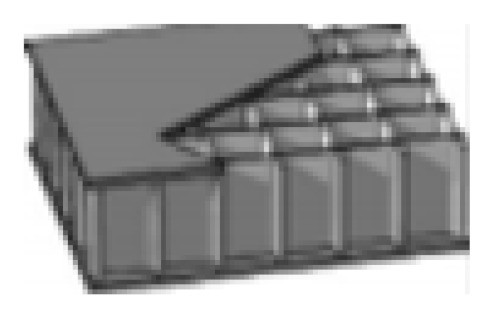	Diamond 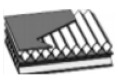	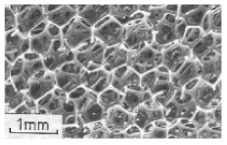	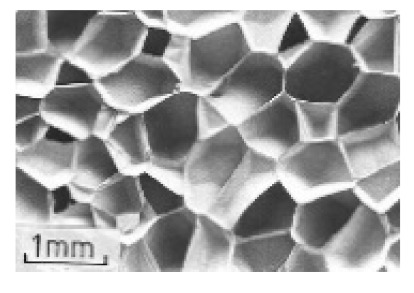
Tetrahedral 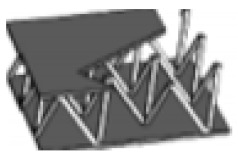	Diamond Textile 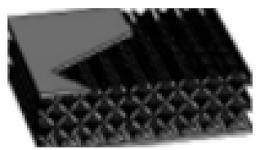	Hexagonal 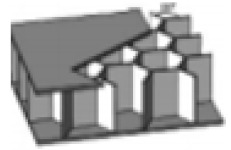	Triangular 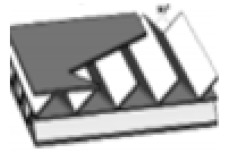		
3D Kagome 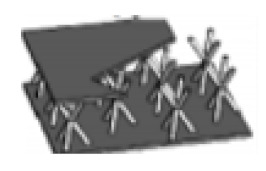	Square Textile 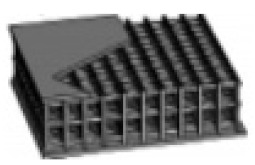	Triangular 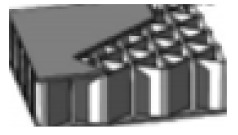	Navtruss 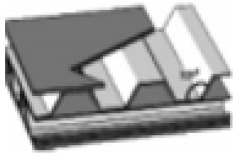		

**Table 3:** Core fabrication techniques.

Type of Materials	Fabricating Process	Parameter Control	Refs.
Synthetic composites	Injection molding	Hexagonal core geometries (lengths of cell walls, wall thickness, and the internal cell angle)	[85]
Synthetic composites	Pre-preg	Geometries	[86]
Synthetic composites	Hand lay-up	Temperature loadings	[87]
		Corresponding thermal strains and stresses	
NFCs	Compression molding process	Core geometries	[88]
NFCs		Material	[89]
Synthetic composite		Process (forming temperature, speed, and the forming angle)	[90]
		Geometry of core	[91]
NFCs	Solidification	Weight	[92]
		Density	[88]
		Geometry	[83]
NFCs	Hot press process	Temperature	[93]
		Pressure	
Synthetic composites		Consolidation time	

**Table 4:** Specific mechanical properties of the sisal–polypropylene core [94].

Material, Cell Size (mm)	Density (kg/m^3^)	σ/ρ(Nm/kg)	τ/ρ(Nm/kg)	E/ρ(Nm/kg)
Sisal–Polypropylene sinusoidal core~12	49 (±12.0)	52.56 × 10^3^	12 × 10^3^	1.82 × 10^6^
Sisal–Polypropylene hexagonal core~12	37 (±3.0)	56.04 × 10^3^	10 × 10^3^	1.73 × 10^6^
Nomex (phenolic)~10	48 (±3.0)	45.83 × 10^3^	27 × 10^3^	2.29 × 10^6^
Aluminum (5056)~10	151 (±7.6)	41.89 × 10^3^	32 × 10^3^	10.54 × 10^6^
Polypropylene core~6	145 (±3.2)	25.27 × 10^3^	8 × 10^3^	0.80 × 10^6^

**Table 5:** Summary of selected papers on natural fiber based core development.

Author	Mechanical Test	Control Parameter	Finding
Stocchi et al. [92]	Compression test Flexural modulus	Design: Honeycomb Compression molding with two molds utilized Fixed inserts (6 mm cell wall) Lateral insert (10 mm cell wall)	Lateral based compression had better results with the capability to produce a thinner wall and remove excess resin, including trapped air bubbles Resulting cores with the same specific compression strength but higher compression strength compared to commercially available counterparts ◦ Specific: 0.085 MPa/Kg and 0.05 MPa/Kg for 6mm and 10mm cell cores, respectively ◦ 6 mm-cell core with 15.5 MPa; 10 mm-cell core with 13.5 MPa Flexural modulus for 10 mm-cell core: Higher dispersion of about 40% than mean value (115.48 ± 1.35 MPa)
Rao et al. [94]	Four-point flexural tests	Sinusoidal core Hexagonal core	Sinusoidal core (6.5 MPa), higher than hexagonal core (5.2 MPa) Static failure stress (40%)
Zuhri et al. [72]	Compression test	Square core Triangular core	Core design and specific strength ◦ 2 mm thickness: square possessed higher strength than triangle cores (flax/PLA) ◦ 2.3 mm thickness: triangle core possessed higher strength (flax/PP) Square structure offered better strength and energy absorption characteristics than triangular structure
Du et al. [98]	Creep test	Relative humidity (20–50%)	Static failure stress (40%)
Chen et al. [104]	Flexural creep test	Expanded core (31.75 mm) Corrugated core (19.05 mm)	Creep flexural deflection rates Expanded honeycomb characterized by higher rates compared to corrugated honeycomb cores
Roslan et al. [102]	Compression test	Triangle core Square core Configuration setup 0-degree orientation 45-degrees orientation 90-degrees orientation	Triangular core superior to its counterpart, with higher energy absorption rates and/or capability, and hence desirable in strength and stability applications.

## Data Availability

The data presented in this study are available on request from the corresponding author.
